# Highly Sensitive Charge Sensor Based on Atom-Assisted High-Order Sideband Generation in a Hybrid Optomechanical System

**DOI:** 10.3390/s18113833

**Published:** 2018-11-08

**Authors:** Zeng-Xing Liu, Hao Xiong

**Affiliations:** School of Physics, Huazhong University of Science and Technology, Wuhan 430074, China

**Keywords:** ultrasensitive charge sensor, optomechanical nonlinearity, high-order sideband generation, phase sensitivity

## Abstract

Realizing highly sensitive charge sensors is of fundamental importance in physics, and may find applications in metrology, electronic tunnel imaging, and engineering technology. With the development of nanophotonics, cavity optomechanics with Coulomb interaction provides a powerful platform to explore new options for the precision measurement of charges. In this work, a method of realizing a highly sensitive charge sensor based on atom-assisted high-order sideband generation in a hybrid optomechanical system is proposed. The advantage of this scheme is that the sideband cutoff order and the charge number satisfy a monotonically increasing relationship, which is more sensitive than the atom-free case discussed previously. Calculations show that the sensitivity of the charge sensor in our scheme is improved by about 25 times. In particular, our proposed charge sensor can operate in low power conditions and extremely weak charge measurement environments. Furthermore, phase-dependent effects between the sideband generation and Coulomb interaction are also discussed in detail. Beyond their fundamental scientific significance, our work is an important step toward measuring individual charge.

## 1. Introduction

Cavity optomechanics exploring the radiation pressure force between the mechanical and optical modes have attracted considerable attention and progressed enormously over the past decades [1,2,3,4,5]. A typical optomechanical system consists of a high-Q Fabry–Perot cavity with one movable mirror that is treated as a mechanical oscillator. This novel subject has created a new perspective to investigate light–matter interaction via radiation–pressure coupling between the mechanical degrees of freedom and the optical cavity mode [3]. The development of cavity optomechanics has opened up avenues for providing a novel platform for many important applications, ranging from the ground-state cooling of mechanical motion [6,7,8] and manipulation of light propagation [9,10,11] to photon (phonon) blocking [12,13,14]. In particular, with the development of nanophotonics, cavity optomechanical systems provide a unique platform to explore new availability for the precision measurement of weak force due to their important properties of small mass and high integrability [15,16,17,18,19,20].

In recent years, investigations of optomechanical nonlinearity [21,22,23] have become a research hotspot, and many interesting phenomena have been revealed. As a fascinating nonlinear phenomenon, high-order sideband generation [24,25,26] is not only of great significance in studying nonlinear characteristics of photon–phonon interaction, but also plays an indispensable role in excavating the potential applications of optomechanical systems. The process of high-order sideband generation is analogous to Stokes and anti-Stokes Raman scattering processes. By pumping the cavity field at frequency $\omega_{1}$, sidebands in the spectrum are created at $\omega_{1}\pm n\Omega_{m}$ due to the radiation–pressure coupling of the photons with the mechanical motion. Here, $\Omega_{m}$ is the angular frequency of the mechanical oscillator and the integer *n* represents the sideband order. Specifically, if the pump is red-detuned such that the upper sideband frequency is matched with the resonance frequency $\omega =\omega_{1}+n\Omega_{m}$, the anti-Stokes scattering is resonantly enhanced. Conversely, if the pump is blue-detuned such that the lower sideband frequency is matched with $\omega =\omega_{1}-n\Omega_{m}$, the Stokes scattering is resonantly enhanced. In addition, the rate of the Stokes and anti-Stokes scattering per phonon is proportional to the number of photons in the optical cavity. Note that the optomechanical nonlinearity originating from radiation–pressure coupling between the cavity mode and the mechanical mode can be substantively strengthened by atomic assistance [27,28]. The physical mechanism can be explained as follows: under specific parameter conditions, stimulated by the two-level atoms trapped in the optical cavity, the photon number in the cavity field will be greatly increased. Simultaneously, the distribution of photon number in the cavity field will be modified, and the radiation pressure coupling between the photon and the mechanical mode will be strengthened. Ultimately, the higher-order sidebands generation output from the cavity field will be greatly reinforced [25].

Some previous studies [29,30] have shown that optomechanical nonlinearity is more sensitive to weak force, which have an attractive application prospect in precision measurement. In Ref. [31] the high-order sideband generation shows a strong dependence on Coulomb interaction, and a proposal of detecting charge based on a Coulomb-interaction-dependent effect is proposed. However, this Coulomb-interaction-dependent effect is non-monotonic and can only been observed when the charge number is large enough, which severely limits the measurement accuracy of the charge sensor. In the present work, we report on a novel detection method that allows us to measure the charge number with an unprecedented sensitivity. Here, two dependent effects of the high-order sideband generation (i.e., Coulomb-interaction-dependent effect [31] and atom-assisted-dependent effect [25]) are combined. We reveal that the high-order sideband generation can not only be dramatically strengthened by atomic assistance, but also reveals a strong dependence on charge number. Furthermore, the sideband cutoff order and the charge number satisfy a monotonically increasing relationship, which is more sensitive than the atom-free case discussed previously. Calculations show that the sensitivity of the charge sensor in our scheme is improved by about 25 times. In particular, based on the current experimental conditions, we believe that the proposed scheme of a highly sensitive charge sensor will be highly accessible in experiments.

## 2. Charge Sensor Model

**Figure 1:**
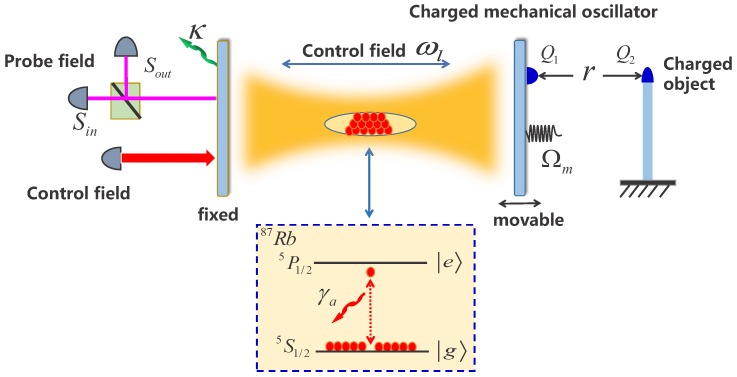
Schematic diagram of a hybrid optomechanical system, in which an ensemble of *N* identical two-level ${}^{87}Rb$ atoms, with transition frequency $\omega_{a}$ and decay rate $\gamma_{a}$, are embedded in the cavity field. Levels |g〉 and |e〉 correspond to the ground state ${|}^{5}S_{1/2}\rangle$ and the excited state ${|}^{5}p_{1/2}\rangle$ of the ${}^{87}Rb$ atom, respectively. The system is driven by a strong control field with frequency $\omega_{l}$ and probed by a weak probe field with frequency $\omega_{p}$. The transmission of the output field can be measured by the homodyne technique [36]. A charged mechanical oscillator with angular frequency $\Omega_{m}$ and charge amount $Q_{1}$ is coupled to an adjoining charged object with charge amount $Q_{2}$ via the Coulomb force.

The charge sensor model is based on a hybrid atom–cavity optomechanical system, as schematically shown in Figure 1, in which one mirror of the cavity is movable and is treated as a mechanical oscillator. *N* identical two-level ${}^{87}Rb$ atoms with the transition frequency $\omega_{a}$ are located within the cavity field, and the Hamiltonian of these two-level atoms can be written as ${\hat{H}}_{\mathrm{atom}}$ = $\hbar \sum_{i=1}^{N}\omega_{a}{\hat{\sigma }}_{ıj}^{\left(i\right)}$, where ${\hat{\sigma }}_{ıj}^{\left(i\right)}$ = ${|ı\rangle }_{\mathrm{ii}}\langle j|$ for ı≠j is the electronic projection operator and ı=j is the population operator of the *i*-th ${}^{87}Rb$ atom. In the present work, we presume that most of two-level ${}^{87}Rb$ atoms are originally prepared in the ground state. Therefore, the average number of the ${}^{87}Rb$ atoms in the excited state is a small fraction of the total ${}^{87}Rb$ atoms number *N*. Under the weak excitation, the condition of the Holstein–Primakoff approximation [32] is satisfied. Therefore, we can define $\hat{ℜ}=\lim_{N\to \infty }\sum_{i=1}^{N}\left(g_{i}^{*}/g_{\mathrm{ac}}\right){|ı\rangle }_{\mathrm{ii}}\langle j|$ as an effective transition operator [33]. $g_{\mathrm{ac}}=g\sqrt{N}=\sqrt{\sum_{i=1}^{N}{\left|g_{i}\right|}^{2}}$ represents the total coupling strength between the atomic en bloc and the cavity field, where *g* is the single-atom vacuum Rabi frequency. The mechanical and cavity modes are coupled as ${\hat{H}}_{\mathrm{int}}$ = $\hbar G{\hat{a}}^{†}\hat{a}\hat{x}$, where *G* is the optomechanical coupling strength and $\hat{a}$ (${\hat{a}}^{†}$) indicates the annihilation (creation) operator of the cavity field. In analogy to nanoelectromechanical systems, the Coulomb interaction can also be introduced to an optomechanical system. A typical configuration is shown in Figure 1, in which a tiny charged object is attached to the mechanical oscillator. In experiment [34,35], an ensemble of nitrogen-vacancy centers and electric charges can be embedded in a single crystal diamond nanobeam. In the same way, an ultra-miniature charged body can also be embedded in the movable mirror of the cavity in the lab. The Hamiltonian of the Coulomb interaction be written as ${\hat{H}}_{\mathrm{elec}}$ = $KQ_{1}Q_{2}\hat{x}/r^{2}$, with K the electrostatic force constant, $Q_{1}$ and $Q_{2}$ respectively the charge number of the mechanical oscillator and the charged body, and *r* the distance between the mechanical oscillator and the charged object. For simplicity, we assume that the charge of the mechanical oscillator $Q_{1}$ is changeless and the variation of $Q_{2}$ can be written as $Q_{2}$ = νe, with *e* the elementary charge and ν the charge number.

In this work, the system is driven by a strong control field with frequency $\omega_{l}$ and a weak probe field with frequency $\omega_{p}$. Applying the rotating wave approximation, viz., $\hbar g_{\mathrm{ac}}\left(\hat{a}+{\hat{a}}^{†}\right)\left(\hat{ℜ}+{\hat{ℜ}}^{†}\right)\to \hbar g_{\mathrm{ac}}\left(\hat{a}{\hat{ℜ}}^{†}+{\hat{a}}^{†}\hat{ℜ}\right)$, the evolutionary dynamics of this hybrid optomechanical system can be well depicted by the Heisenberg–Langevin equations as:

(1) $$\begin{array}{ccc} \dot{\hat{x}} & = & \hat{p}/m,\\ \dot{\hat{ℜ}} & = & -\left(\gamma_{a}+i\Delta_{a}\right)\hat{ℜ}-ig_{\mathrm{ac}}\hat{a}+\sqrt{2\gamma_{a}}{\hat{\sigma }}_{th}\left(t\right),\\ \dot{\hat{p}} & = & -m\Omega_{m}^{2}\hat{x}-\hbar G{\hat{a}}^{†}\hat{a}-\gamma_{m}\hat{p}-kQ_{1}Q_{2}/r^{2}+\sqrt{2\gamma_{m}}{\hat{\Gamma }}_{th}\left(t\right),\\ \dot{\hat{a}} & = & -\left[2\kappa +i\left(\Delta_{c}+G\hat{x}\right)\right]\hat{a}-ig_{\mathrm{ac}}\hat{ℜ}+\varepsilon_{l}+\varepsilon_{p}e^{-i\delta t}+\sqrt{2\kappa }{\hat{\Upsilon }}_{th}\left(t\right), \end{array}$$

where $\hat{p}$ ($\hat{x}$) is the momentum (position) operator of the mechanical oscillator with the effective mass *m* and angular frequency $\Omega_{m}$. $\Delta_{c(a)}$ = $\omega_{c(a)}-\omega_{l}$ is the detuning of the cavity field and the two-level atoms with respect to the driving field, respectively. $\varepsilon_{i}=\sqrt{2\kappa P_{i}/\hbar \omega_{i}}\;\left(i=l,p\right)$ are the amplitudes of the input field with $P_{l}$ the power of the control field, and $P_{p}$ the power of the probe field. κ = $\kappa_{o}$ + $\kappa_{ex}$ is the total decay rate of the cavity field containing an intrinsic loss rate $\kappa_{o}$ and an external loss rate $\kappa_{ex}$ [3]. The decay rates of the mechanical oscillator $\gamma_{m}$ and the two-level atom $\gamma_{a}$ are introduced classically. The thermal noise of the mechanical oscillator ${\hat{\Gamma }}_{th}\left(t\right)$ satisfies the correlation function $\langle {\hat{\Gamma }}_{th}\left(t\right){\hat{\Gamma }}_{th}^{†}\left(t\right)\left(t^{\prime }\right)\rangle =\gamma_{m}\int e^{-i\omega (t-t^{\prime })}\left[\coth\left(\hbar \omega /2k_{B}T\right)+1\right]d\omega /2\pi \Omega_{m}$ [37], with $k_{B}$ the Boltzmann constant and *T* the temperature of the mechanical oscillator. Additionally, the quantum noise of the cavity field ${\hat{\Upsilon }}_{th}\left(t\right)$ and the two-level atom ${\hat{\sigma }}_{th}\left(t\right)$ satisfy the correlation function $\langle {\hat{\Upsilon }}_{th}\left(t\right){\hat{\Upsilon }}_{th}^{†}\left(t^{\prime }\right)\rangle =\delta \left(t-t^{\prime }\right)$, $\langle {\hat{\sigma }}_{th}\left(t\right){\hat{\sigma }}_{th}^{†}\left(t^{\prime }\right)\rangle =\delta \left(t-t^{\prime }\right)$, respectively. Here, under the semiclassical approximation, the quantum noise can be dropped safely because their mean values are zero (i.e., $\langle {\hat{\Gamma }}_{th}\left(t\right)\rangle =0$, $\langle {\hat{\Upsilon }}_{th}\left(t\right)\rangle =0$, and $\langle {\hat{\sigma }}_{th}\left(t\right)\rangle =0$). Here, we need to point out that the photothermal forces [38] in our scheme can be safely ignored because the delay time of the photothermal forces is much larger than the measurement time of the sideband spectrum in the laboratory [9]. All the operators can be reduced to their expectation values, that is, $x\left(t\right)\equiv \langle \hat{x}\left(t\right)\rangle$, $p\left(t\right)\equiv \langle \hat{p}\left(t\right)\rangle$, $a\left(t\right)\equiv \langle \hat{a}\left(t\right)\rangle$, and ${ℜ}_{a}\left(t\right)\equiv \langle {\hat{ℜ}}_{a}\left(t\right)\rangle$, because we focus on the average response of the system [1]. Using mean-field approximation, viz., 〈xa〉=〈x〉〈a〉, the Heisenberg–Langevin equations can be written as:

(2) $$\begin{array}{ccc} \dot{x} & = & p/m,\\ \dot{ℜ} & = & -\left(\gamma_{a}+i\Delta_{a}\right)ℜ-ig_{\mathrm{ac}}a,\\ \dot{p} & = & -m\Omega_{m}^{2}x-\hbar Ga^{*}a-\gamma_{m}p-kQ_{1}Q_{2}/r^{2},\\ \dot{a} & = & -\left[2\kappa +i\left(\Delta_{c}+Gx\right)\right]a-ig_{\mathrm{ac}}ℜ+\varepsilon_{l}+\varepsilon_{p}e^{-i\delta t}. \end{array}$$

Equation (2) is a group of partial differential equations that can be numerical solved by using the Runge–Kutta method. The output field $s_{\mathrm{out}}\left(t\right)$ can be obtained by using the standard input–output notation $s_{\mathrm{out}}\left(t\right)=s_{\mathrm{in}}\left(t\right)-\sqrt{2\kappa }a\left(t\right)$, where $s_{\mathrm{in}}\left(t\right)$ are the effective driven fields. The output spectrum S(ω) in the frequency domain can be acquired by performing the fast Fourier transform of $s_{\mathrm{out}}\left(t\right)$, that is, $S\left(\omega \right)\propto |\int_{-\infty }^{\infty }s_{out}\left(t\right)e^{-i\omega t}dt|$, where ω is the spectrometer frequency of the output field [24]. The physical picture of this transform is that the output field of the system in the time domain can be formally expressed by $s_{\mathrm{out}}\left(t\right)=\sum_{j=0}^{n}A_{n}e^{-i(\omega_{1}\pm n\delta )t}$(n=0,1,2···), where $A_{n}$ is the *n*-th transmission coefficient of the output field. After the fast Fourier transform [39], the frequency spectrum of the output field will generate a component at frequencies $\omega_{1}\pm n\delta$ in the frequency domain where *n* is referred to as the sideband order.

Note that if $g_{\mathrm{ac}}=0$, our system is reduced to a traditional optomechanical system with Coulomb interaction, and a proposal for detecting charge based on a Coulomb-interaction-dependent effect has been proposed [31]. Under weak driven fields (about a few mW), the variation of the sideband spectrum is about 11.83% per charge, and the relationship between the sideband order and the charge number is non-monotonic. In this context, we propose a novel scheme for the precision measurement of charge number based on atom-assisted high-order sideband generation in a hybrid optomechanical system, which allows us to measure the charge number with an unprecedented sensitivity. In our scheme, a monotonic relationship between the cutoff order of the sideband spectrum and the charge number is obtained, and the variation of the sideband spectrum is about 300% per charge. Calculations show that the sensitivity of the charge sensor is improved by about 25 times with respect to the atom-free case discussed previously.

## 3. Results and Discussion

In what follows, we discuss the relationship between the atom-assisted high-order sideband generation and the Coulomb interaction, and a feasible method for achieving highly sensitive charge sensor is proposed. First, it is necessary to evaluate the actual experimental possibilities of such a highly sensitive charge sensor. In the experiment, we chose a microscale optomechanical system with a size of about 50 μm, mass of the mechanical oscillator *m* = 10 ng, and half cavity linewidth κ/2π = 1 MHz. An ensemble of identical ${}^{87}$Rb atoms were trapped in the cavity field. The amount of charge on the charged vibrator was $Q_{1}$ = 27.5 nC and the distance between the charged body and the charged vibrator was r = 67 μm. The power of the control field $P_{l}$ = 0.5 mW and the weak probe field with probe power $P_{p}$ = 1.25 μW, and the transmission of the output field could be measured by the homodyne technique. All these conditions already have mature experimental foundations [6,34,40], so the proposed scheme of a highly sensitive charge sensor will be highly accessible in the laboratory.

**Figure 2:**
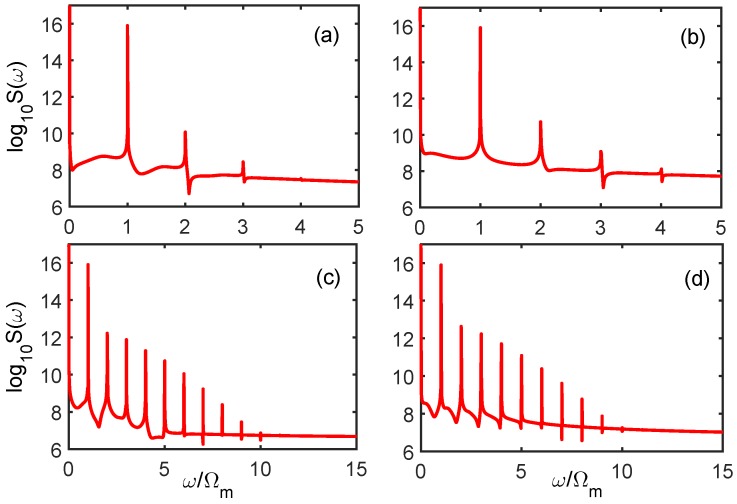
The high-order sideband spectra output from the optomechanical system in the absence of two-level atoms, shown with various charge numbers *n*: (**a**) *n* = 50; (**b**) *n* = 100; (**c**) *n* = 150; and (**d**) *n* = 200. The parameters were *m* = 10 ng, $\Omega_{m}/2\pi$ = 10 MHz, G/2π = 0.4 GHz/nm, κ/2π = 1 MHz, $\gamma_{m}/2\pi$ = 100 Hz, $g_{\mathrm{ac}}/6\pi$ = 4.2 MHz, $\gamma_{a}/2\pi$ = 2.875 MHz, and *r* = 67 μm, K = $8.99\times \;10^{9}$ N· m${}^{2}$/C${}^{2}$, $Q_{1}$ = CU with *C* = 27.5 nF, *U* = 1 *V*, $\Delta_{c}=\Delta_{a}=\Omega_{m}$, $P_{l}$ = 0.5 mW.

Figure 2 plots the high-order sideband generation output from the cavity field under different charge number *n*. In the absence of two-level atoms, the optomechanical nonlinearity is quite weak so that the anti-Stokes scattering processes are severely suppressed. As shown in Figure 2a, the sideband ended up at the order of 3 when the charge number n=50. In Figure 2b, even though the charge number *n* increased to 100, the cutoff order and the amplitude of the sideband spectrum were hardly enhanced. Obviously, it is very difficult to obtain a strong dependency between the high-order sideband generation and the Coulomb interaction when the charge number is not large enough. Upon further increase of the charge number *n* to 150, as Figure 2c shows, the cutoff order of the sideband spectrum was extended to about 10 and the amplitude of each sideband was also obviously enhanced. A similar result is shown in Figure 2d, when we continued to increase the number of charges to n=200. From the above discussion, we can see that the Coulomb-interaction-dependent effect without atomic assistance is far from sufficient for the precision measurement of charge number, and is unsuitable for extremely weak charge measurement environments.

**Figure 3:**
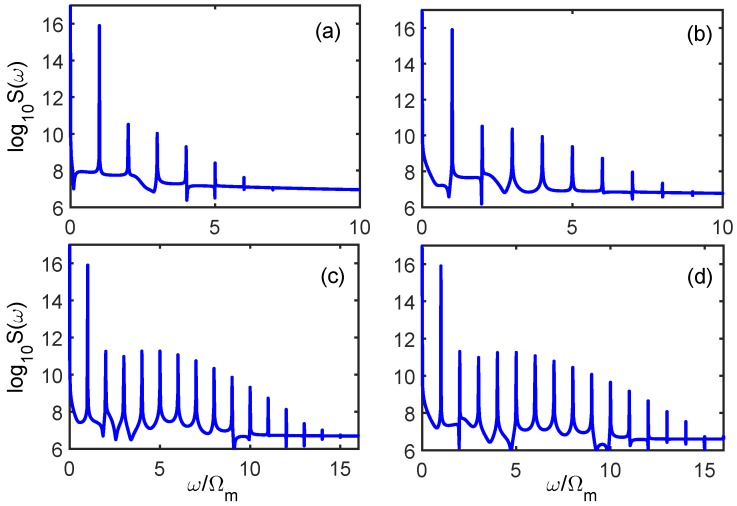
The high-order sideband spectral output from the optomechanical system in the presence of the two-level atoms are shown with various charge numbers *n*: (**a**) *n* = 1; (**b**) *n* = 2; (**c**) *n* = 3; and (**d**) *n* = 4. The other parameters are the same as those in Figure 2.

Previous studies have shown that resonantly enhanced feedback-backaction arising from optomechanical nonlinearity can be substantively reinforced by atomic assistance [27,28]. A strong dependence between the high-order sideband generation and the charge number *n* is observed in Figure 3. In contrast to Figure 2a, both the cutoff order of the sideband spectrum and the amplitude of each sideband were markedly enhanced owing to the reinforcement of optomechanical nonlinearity between the cavity field and the mechanical oscillator. As shown in Figure 3a, the higher-order sidebands ended up at the order of 6 and the intensity of each sideband was also strengthened. Intriguingly, high-order sideband generation in such atom–cavity optomechanical systems has a strong dependence on the charge number. As shown in Figure 3b, when a single charge was added to the charged body (charge number *n* = 2), we can clearly see that the order of the sidebands ended up at the order of 8, and the intensity of each sideband was also distinctly enhanced. More robust high-order sideband generations are shown in Figure 3c,d, respectively, when the charge numbers *n* = 3 and 4 are considered. Obviously, not only did the cutoff order extend to 14 and 15 corresponding to the charge numbers *n* = 3 and 4, but the amplitude of the sideband spectra were increased significantly as well. In addition, the sideband spectra exhibiting a typical non-perturbative nature were quite different from Figure 3a,b. That is, the intensity of the lower-order sidebands were smaller than the higher-order sidebands, which indicates that the strong optomechanical nonlinearity was greatly reinforced by atomic assistance. Comparing Figure 2 and Figure 3, the ultra-sensitive Coulomb-interaction-dependent effect of high-order sideband generation reminds us of the possibility of realizing a highly sensitive charge sensor using an atom-assisted optomechanical system.

**Figure 4:**
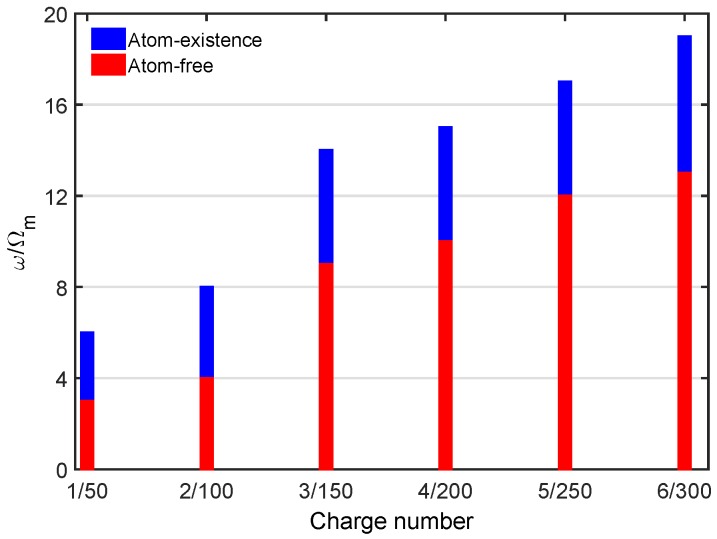
The cutoff order of the higher-order sidebands varied with charge number *n* under two circumstances: the atom-existence case (blue columns) and the atom-free case (red columns). The other parameters are the same as those in Figure 2.

**Figure 5:**
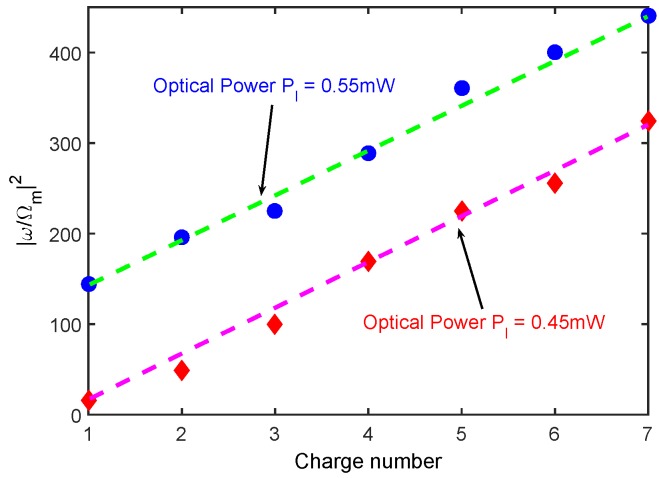
The relationship between the charge number *n* and the square value of the sidebands cutoff order $|\omega /\Omega_{m}{|}^{2}$ in the presence of two-level atoms under different optical power. The other parameters are the same as those in Figure 2.

Figure 4 plots the cutoff order of the high-order sideband varying with the charge number *n* under the atom-existence case and the atom-free case, respectively. As the red columns show in Figure 4, the cutoff order of the higher-order sidebands was almost unchanged when the number of charges was less than 100. Only when the charge number was sufficient (about 150), the cutoff order of the higher-order sidebands had obvious changes. In our scheme, as the blue columns show, a clear change in the cutoff order of the sideband spectrum was observed, even though a single charge was embedded in the charged object, which provides us with the possibility of proposing the precision measurement of single charges. Additionally, we note that the dependence of the cutoff order of the high-order sideband generation on the charge number had a monotonous relationship. As shown in Figure 5, the monotonous relation between $|\omega /\Omega_{m}{|}^{2}$ and the charge number *n* held for different optical powers. This monotonous relationship for the cutoff order of the sideband spectrum in the presence of electric interaction is more convenient and useful than the non-monotonous relationship discussed previously [31].

**Figure 6:**
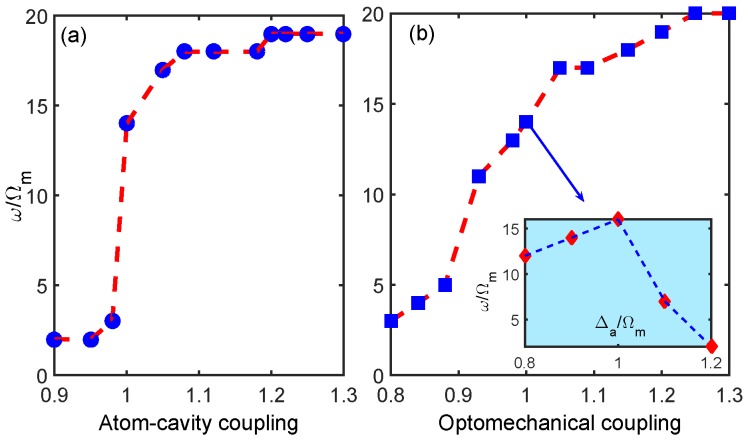
The cutoff order of the higher-order sidebands as a function of (**a**) the atom–cavity coupling strength $g_{\mathrm{ac}}/g_{\mathrm{ac}0}$ ($g_{\mathrm{ac}0}/6\pi$ = 4.2 MHz) and (**b**) the optomechanical coupling strength $G/G_{0}$ ($G_{0}/2\pi$ = 0.4 GHz/nm). Inset: The cutoff order of the higher-order sidebands as a function of the atomic detuning $\Delta_{a}/\Omega_{m}$. The other parameters are the same as those in Figure 2.

In order to investigate the influences of other degrees of freedom of the system on the sideband sensitivity change, the cutoff order of the sideband spectrum as a function of the atom–cavity coupling $g_{\mathrm{ac}}$, the optomechanical coupling *G*, and the atomic detuning $\Delta_{a}$, respectively, are plotted in Figure 6. We observed that in the case of weak atom–cavity coupling strength (i.e., $g_{\mathrm{ac}}/g_{\mathrm{ac}0}<1$), the order of the sidebands did not increase significantly. However, as the atom–cavity coupling strength increased, the order of sidebands increased sharply, and then tended to smooth. The physical mechanism is that the total coupling strength between the atomic en bloc and the cavity field is determined by the number of the two-level atoms (i.e., $g_{\mathrm{ac}}=g\sqrt{N}$). In the case of a small number of atoms, the optomechanical nonlinearity cannot be significantly enhanced so that the generation of the high-order sideband is not obvious. As the number of atoms increases, the photon distribution of the cavity field changes, as well as the radiation pressure coupling between the photon and the mechanical mode will be strengthened. As expected, the sideband effect was conspicuously enhanced, as shown in Figure 6a. Upon continuing to increase the number of atoms, however, the sideband effect was no longer strengthened because the system entered the over-coupling regime and the nonlinear strength tended to be saturated. Similar results are shown in Figure 6b. As we increased the optomechanical coupling strength, the cutoff order of the sideband spectrum was observed to transit from a slow increase state to a region of rapid increase, finally entering the stationary regime. All these results agree very well with those of previous studies [25]. The inset in Figure 6b plots the cutoff order of the higher-order sidebands as a function of the atomic detuning $\Delta_{a}/\Omega_{m}$. We noted that the maximum sideband effect appeared if and only if the detuning of the atoms $\Delta_{a}$ was matched with the resonance frequency $\omega_{a}/\Omega_{m}=1$.

**Figure 7:**
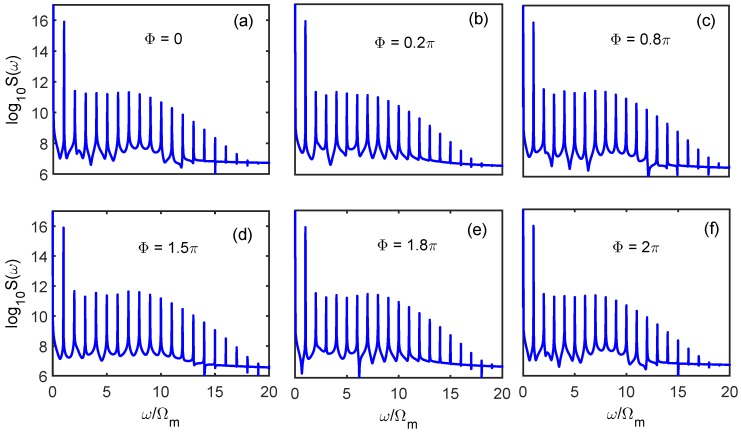
The high-order sideband spectra output from the hybrid atom–cavity optomechanical system with electric interaction (the charge number n=5) are shown with different phase: (**a**) Φ=0π; (**b**) Φ=0.2π; (**c**) Φ=0.8π; (**d**) Φ=1.5π; (**e**) Φ=1.8π; (**f**) Φ=2π. The other parameters are the same as those in Figure 2.

Finally, phase-dependent effects of the high-order sideband generation with electric interaction were investigated, which remains largely unexplored and may provide applications in phase-coherent measurement. We started with the Hamiltonian of the probe field $H_{\mathrm{probe}}$ = $\varepsilon_{p}^{*}{\hat{a}}^{†}e^{-i(\omega_{p}t+\Phi )}+H.C.$ Here, Φ is the relative phase of the two input lasers, that is, Φ = $\Phi_{p}-\Phi_{l}$ with $\Phi_{p}$ the initial phase of the probe field and $\Phi_{l}$ the initial phase of the driving field [41]. Figure 7 shows the high-order sideband generation output from the cavity field under different phase Φ. Here, we chose the charge number *n* = 5 for the purpose of obtaining a robust sideband spectrum. From Figure 7 we can see that the relative phase of the two input lasers exhibited a certain influence on the sideband spectrum output from the cavity field. When we chose the relative phase Φ = 0.2 π, the amplitude of each sideband was weaker than the circumstances without considering the phase effect. However, as shown in Figure 7c, the intensity of each sideband amplitude was obviously enhanced. Physically, different phases will give rise to destructive interference or constructive interference processes, which corresponds to suppressing or enhancing the optomechanical nonlinearity of the system. The intensity of the sideband amplitude is ultimately reflected in the output spectrum. Phase is an important information carrier, and discussing phase-dependent effects may help to explore the coherence properties of photons. Therefore, our results may have potential applications in phase-coherent measurement and optical communications, as Refs. [42,43,44,45] recommend.

## 4. Conclusions

To conclude, we demonstrated that the atom-assisted optomechanical system can be employed as a charge sensor with an unprecedented sensitivity. We observed that a strong dependency between the high-order sideband generation and the Coulomb interaction could be greatly reinforced by atomic assistance, and a monotonous relationship between the cutoff order of the sideband spectrum and the charge number was obtained. In addition, the influences of system degrees of freedom on the sidebands generation (e.g., the atom–cavity coupling, the optomechanical coupling, and the atomic detuning) were investigated in detail. Compared to previous charge sensing proposals, our scheme proposed here has some obvious advantages. First, our scheme is expected to achieve accurate detection at the level of single charges because the variation of the sideband spectrum is about 300% per charge, which is improved by about 25 times with respect to the atom-free case discussed previously. Second, our solution is suitable for extremely weak charge measurement environments. A robust Coulomb-interaction-dependent effect arises as long as the charged object has a charge, rather than requiring that the amount of charges exceed approximately 30. Third, the power of the control field, about 0.5 mW, is quite weak. Therefore, our proposed charge sensor can operate in a low-power environment. Finally, we found that the effective adjustment of the sideband spectrum could also be implemented via phase manipulation, which may deepen our understanding of optomechanical nonlinear processes and may provide applications in phase-coherent measurement. Using the current experimental parameters, our results show that atom-assisted high-order sideband generation may provide a novel method to achieve a highly sensitive charge sensor.
